# Using a large citizen science dataset to uncover diverse patterns of elevational migration in Himalayan birds

**DOI:** 10.1098/rsos.242260

**Published:** 2025-05-07

**Authors:** Tarun Menon, Paul R. Elsen, Umesh Srinivasan

**Affiliations:** ^1^ Centre for Ecological Sciences, Indian Institute of Science, Bangalore, Karnataka, India; ^2^ Global Conservation Program, Wildlife Conservation Society, Bronx, NY, USA

**Keywords:** citizen science, elevational movement, Himalayas, eBird, guilds, partial migration

## Abstract

Among montane birds, elevational migration is a well-described phenomenon. Yet, apart from mountain ranges in the Americas, there is little information on the large-scale patterns and extent of elevational migration. Using a large citizen science dataset (eBird), we determine the elevational ranges of 377 Himalayan bird species in their breeding and non-breeding periods. Based on the position of species’ seasonal elevation ranges, we describe five elevational migration patterns that broadly include post-breeding upslope and downslope migration. Most high-elevation breeders (65–75%) were downslope migrants, which were further subdivided into four distinct patterns: ‘displace’ (complete downslope), ‘shift’ (partial downslope), ‘expand’ (lower limit expansion) and ‘contract’ (upper limit contraction). We find significant intraspecific variation in migration patterns across the Himalayas, possibly determined by local biotic and abiotic conditions. Specialized dietary guilds like invertivores were more likely to show shift or displace migration, potentially tracking seasonally fluctuating food resources, while generalists like omnivores and human commensals were more likely to be resident. Territorial birds were largely non-migratory, most likely to retain high-quality breeding territories. As mountains are a bounded domain with limited combinations of species’ seasonal elevation ranges, the patterns we describe here are useful for understanding elevational migration globally.

## 1. Introduction

Most of the world’s bird diversity is concentrated in tropical mountains where elevational migration—the seasonal movement of populations across an elevation gradient—is a common phenomenon. Despite seasonal elevational migration being a dominant life history strategy in montane birds (20–70% of montane avifauna in different mountain ranges [1]), little is known about their migratory patterns and how these might correlate with various life history and functional traits [2]. Almost 20% of all birds migrate, of which half migrate elevationally (i.e. 10% of all birds), yet the overwhelming majority of bird migration studies focus on long-distance latitudinal migration [3,4]. Long-distance latitudinal migration is generally considered obligate, characterized by regularity, consistency and predictability [1,5,6], notwithstanding examples of latitudinal partial migration [7,8]. By contrast, elevational migration has often been described as short-distanced and, at times, facultative, where not all individuals of a population may move (also known as partial migration [1,5,9,10]).

In neotropical mountains, many bird species (mostly frugivores and nectarivores) move upslope to exploit fruiting and flowering resources available at high elevations during the dry season. During the wet season, the same birds move down to lower elevations to escape extreme precipitation events and tropical storms that impede their ability to forage despite the availability of resources [11,12]. Unlike tropical mountains, sub-tropical and temperate mountains experience significant seasonal variation in temperature with cold winters and warm summers and hence patterns and drivers of migration are different.

The Himalayas are a sub-tropical mountain range containing many of the world’s highest peaks. This massive elevation gradient gives rise to a variety of climates, from tropical-like climate in the foothills, to tundra and permanent snow and ice at the highest elevations. Birds in the Himalayas can be found breeding up to 6000 m.a.s.l. [13]. Higher elevations in the Himalayas are prone to sub-zero temperatures and so a large proportion (approx. 70%) of birds breeding at these elevations are expected to migrate elevationally to some degree [14,15].

The Himalayas also span approximately 2000 km along an east-west axis, with strong gradients in temperature/precipitation seasonality and species richness. The eastern Himalayas have almost twice the number of bird species and are approximately half as seasonal compared with the western Himalayas [16], with differences greatest at low elevations (<1000 m). High-elevation habitats (>2500 m) have more similar communities across the Himalayas [16]. It is likely that most birds that breed above 2500 m are elevational migrants, but the migration patterns of the same species might differ across the Himalayas due to differences in temperature seasonality and species richness, which are also known to drive species range limits through thermal tolerance and interspecific competition [17].

Here, we used a large citizen science database (eBird) to estimate species’ seasonal upper and lower elevational range limits, thus attempting to (i) elucidate the diversity of elevational migration patterns exhibited by Himalayan birds based on the variation in their seasonal elevational distributions, (ii) assess whether species exhibit different migration patterns across a temperature seasonality and species richness gradient, and (iii) determine whether functional traits could explain these visible elevational migration patterns at a regional and Himalayas-wide scale.

We considered four functional traits in our analysis: (i) *Diet:* We expected the food availability hypothesis to predict that species dependent on climate-sensitive resources, such as arthropods [18,19], fruit [20] and seeds [21], to migrate downslope to a greater degree than species with more generalist diets that can more readily switch between food resources seasonally, eliminating the need to migrate [22]. (2) *Habitat:* We predicted, based on the climatic constraint hypothesis, that species that breed in more open habitats are more likely to migrate downslope because open habitats have little to no climatic buffering and thus species would need to migrate to avoid pronounced temperature, precipitation or wind exposure and fluctuations compared with forests where intra-annual climatic variation is attenuated [23]. Species associated with human-modified habitats may be subsidized by human resources and would also be less likely to migrate [24–26]. (3) *Territoriality:* We expected, based on the arrival time hypothesis, territorial species to be more sedentary or move much shorter distances because competition for high-quality breeding territories may cause species to either guard these territories year-round or remain nearby in winter so that they are the first to establish their territories in the summer [27,28]. (4) *Body size:* We predicted that larger birds were more likely to be resident and smaller birds more likely to be migrants because the thermal tolerance hypothesis suggests that larger endotherms are less limited by colder temperatures than smaller endotherms [29,30].

## 2. Methods

### 2.1. Data and data curation

**Figure 1.**
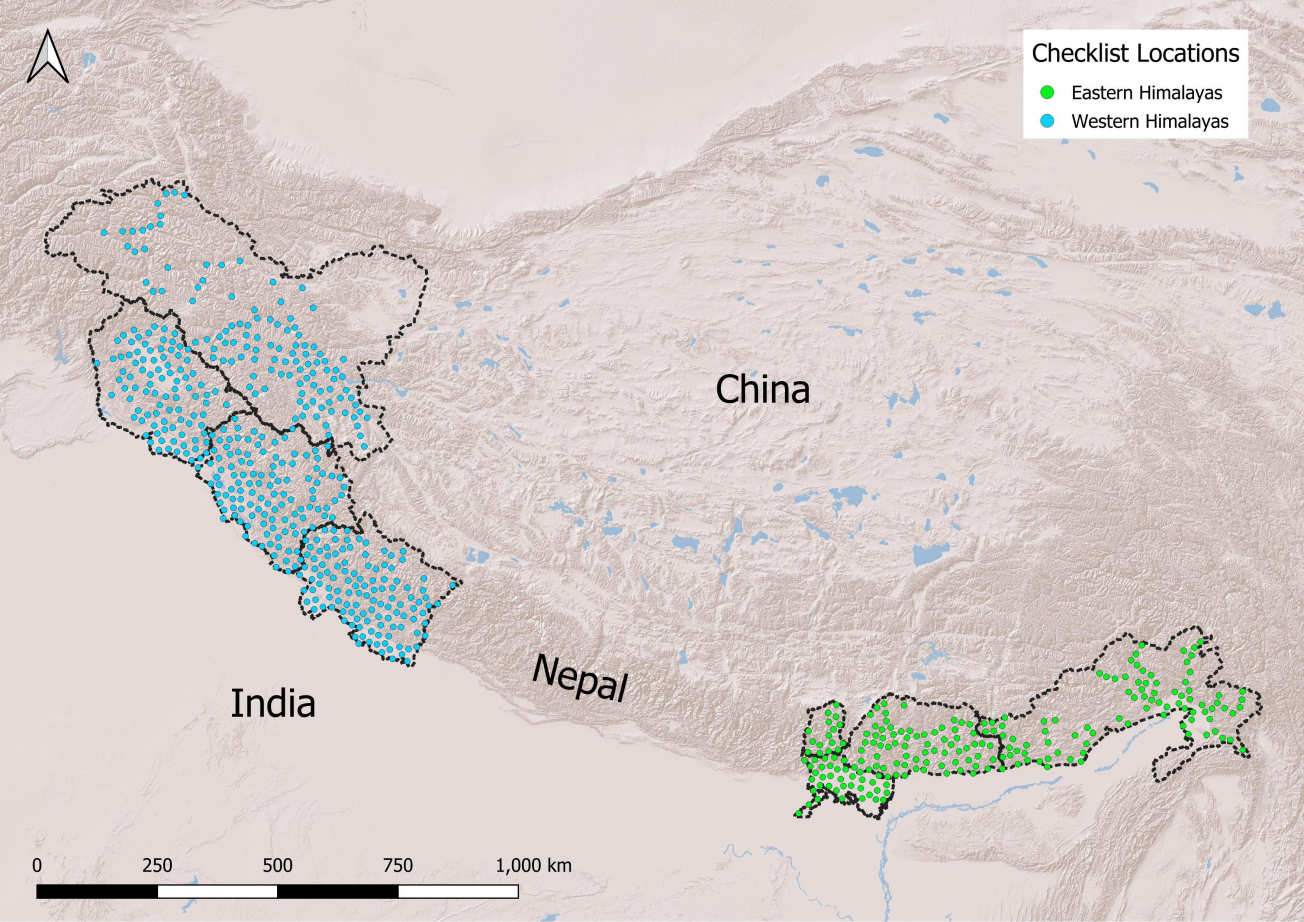
Map showing eBird checklist locations in the Himalayas. The dotted lines represent the political boundaries of the states and countries used to demarcate the eastern (Sikkim, Northern region of West Bengal, Bhutan, Arunachal Pradesh) and western (Jammu and Kashmir, Ladakh, Himachal Pradesh, Uttarakhand) Himalayas. For ease of visualization, we spatially thinned checklists to show only 657 unique locations, by maintaining a minimum distance of 15 km between each checklist location.

eBird is a large citizen science initiative that allows people from around the world to upload checklists of birds they have observed at specific locations [31]. We downloaded eBird observations up to April 2024 from Bhutan and the Indian states of Arunachal Pradesh, Sikkim, West Bengal, Uttarakhand, Himachal Pradesh and the union territories of Jammu and Kashmir and Ladakh (figure 1). We excluded Nepal so that the eastern and western Himalayas were more definitively delineated, and observed patterns at either end were not dampened [16,17,32,33]. Additionally, the data for Nepal are quite sparse (<10 000 checklists that match our requirements), with most checklists (70%) from one single province, which prevented us from creating a separate central region. We considered only ‘complete’ checklists where all birds detected by an observer were reported [34]. We further subset the data to only include checklists where the observer had travelled less than 2.5 km and was birdwatching for less than 120 min to ensure accuracy of elevation data. We extracted the elevation associated with each checklist using the SRTM Digital Elevation Model [35]. We derived data on morphology, dietary guilds and habitat guilds from the AVONET database [4], containing comprehensive eco-morphological data for >95% of all bird species. Information on territorial behaviour came from Tobias *et al*. [36], which contains derived data on territoriality from a comprehensive review of literature and online resources.

### 2.2. Estimating seasonal elevation ranges

We considered checklists from May to August to be breeding (summer) and December to February to be non-breeding (winter) seasons. The sampling effort (number of checklists) was uneven across elevations (biased low), which may cause the naïve estimate of the elevational distribution of a species to be skewed towards the lower elevations. Using a resampling protocol originally described by Tsai *et al*. [37] and modified for a Himalayan dataset by Menon *et al*. [15], we produced an estimate of a species’ elevational distribution in each season and region. eBird checklists from the Himalayas were pooled into seven 500 m elevation bands up to 3000 m; all checklists above 3000 m were pooled together. We resampled checklists with replacement and made a sampling effort (number of checklists) that was equal across every combination of elevation band and season. This was repeated for three different levels of sampling effort, which correspond to the first, second and third quartiles of sampling effort among elevation bands and season (east: 789, 1271, 1791; west: 1736, 3589, 4231). We calculated the median elevation that a species was recorded at in each season to represent the centre of the species’ seasonal elevational distribution. For a species’ upper and lower seasonal elevational distribution limits, we similarly calculated the 95th and 5th percentiles of all elevations that a species was recorded at per season. Resampling was then repeated 1000 times to produce an estimate (and an associated 95% confidence interval) of the centre, upper and lower seasonal elevational limits for each species. This produces 1000 sets of resampled checklists for each of the three levels of sampling effort in the east and west. We see that the difference in the estimated elevation limits between the first and third quartile was very low and below 100 m in most cases (electronic supplementary material, figure S1). Therefore, in the subsequent analysis, we only used the resampled checklist dataset produced from median sampling effort (east: 1271 and west: 3589). Due to the low number of detections of certain rare species, we may estimate elevational ranges that have a high degree of uncertainty, and this is likely to result in a systematic bias towards categorization as a resident. So, from each resampled set, we only analyse species with at least 30 detections across the entire elevational gradient in either season. Additionally, we also conducted a logistic regression to test whether the likelihood of a species being classified as resident varies as a function of sample size or confidence interval size (we found no relationship in either the eastern or western Himalayas; electronic supplementary material, tables S1,S2). We excluded raptors (Accipitriformes), swifts (Apodiformes), swallows (Hirundinidae) and falcons (Falconidae), because observations of these birds are generally made when they are in flight and estimating elevation might be prone to error. Populations that make large latitudinal movements can simultaneously move elevationally, but this is beyond the scope of our study. We therefore restrict our study to one dimension and only include species or populations that breed in the Himalayas and move along the Himalayan elevational gradient [13,38,39].

### 2.3. Description of elevation migration patterns

**Figure 2.**
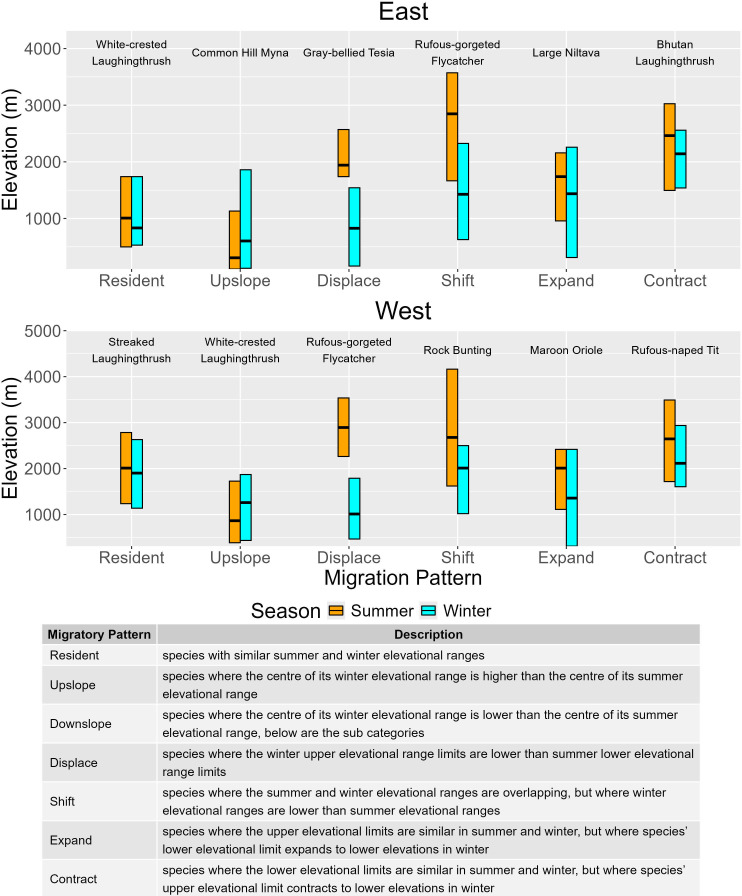
Representative examples of species showing each migration pattern (defined again in the table below) in the eastern and western Himalayas. Each bar represents the species’ estimated upper limit, lower limit and centre (black bar) of their elevational distribution in each season.

Based on the seasonal elevation ranges of all species, we describe five distinct patterns of elevational migration in Himalayan birds, in addition to species that did not migrate seasonally (figure 2). We considered a species to be ‘resident’ if, across seasons, the 95% confidence interval of the centre or both limits (upper and lower) of a species’ elevation distribution (obtained from the 1000 resamples) overlapped. Species whose difference in the estimated centre of elevation distribution between seasons was less than 200 m were also considered as residents. This was a subjective choice informed by our understanding of Himalayan birds that balances using a strict threshold of 0 m (implausible given the range over which a bird may move within a season and the spatial precision of eBird data; [34]) and a more lenient threshold of 500 m (a distance over which many migrants are known to move; [14]).

‘Downslope’ migration was defined as a post-breeding movement of species from higher elevation breeding grounds to lower elevations in the winter. We considered a species to be a downslope migrant if the centre of a species’ elevational distribution is more than 200 m lower in the winter compared with the summer and the 95% confidence intervals do not overlap. We subdivided downslope migration into four distinct types, the naming convention used to describe the type of movement was with respect to a species’ summer range:

(1) ‘Displace’ migration is when the summer and winter ranges of a species are non-overlapping and where the lower limit of a species’ summer distribution is higher than the upper limit of its winter distribution. The centre of a species’ elevational distribution in the winter was on average 1524 m (±893) lower in the east and 2153 m (±331) lower in the west. Species in this landscape can take up to a month to fully occupy their breeding/wintering ranges [40]; so, species that move the most are more likely to have their lower summer limit or upper winter limit extended due to the detection of stragglers or birds in passage. We therefore relaxed the requirement for no overlap between the lower limit of a species’ summer distribution and the upper limit of its winter distribution to an overlap of less than 10% of the species’ overall range.
(2) ‘Shift’ migration was observed when the lower and upper limits of a species’ elevation distribution are more than 200 m lower in the winter and the 95% confidence intervals do not overlap. The centre of a species’ elevation distribution in the winter was on average 937 m (±109) lower in the east and 986 m (±127) lower in the west. What differentiates it from displace migration is the large overlap (>10% of the overall range) between the lower limit of a species’ summer distribution and the upper limit of its winter distribution.
(3) ‘Expand’ migration was observed when the upper limit of a species’ elevation distribution changed by less than 200 m across seasons or there was an overlap in the 95% confidence intervals. The lower limit of the species’ elevation distribution is more than 200 m lower in the winter and the 95% confidence intervals do not overlap. This was on average 392 m (±71) lower in the east and 416 m (±96) lower in the west. Due to intraspecific variation in migration propensity, some individuals may overwinter at their high-elevation breeding grounds while some migrate, expanding the species’ range downslope in the winter.
(4) ‘Contract’ migration was observed when the upper limit of the species’ elevation distribution is more than 200 m lower in the winter and the 95% confidence intervals do not overlap. This was on average 560 m (±89) lower in the east and 805 m (±194) lower in the west. However, the lower limit of a species’ elevation distribution changed by less than 200 m across seasons or there was an overlap in the 95% confidence intervals. Again, due to intraspecific variation in migration propensity, some individuals of the species that breed in the lower part of their elevational range overwinter at the same elevations, while those breeding at higher elevations migrate, contracting the species’ range downslope in the winter.

Finally, we also documented ‘upslope’ migration, which was defined as a post-breeding movement of species from lower elevation breeding grounds to higher elevations in the winter. We considered a species to be an upslope migrant if the centre of a species’ elevational distribution is more than 200 m higher in the winter and the 95% confidence intervals do not overlap. The centre of a species’ elevational distribution was on average 311 m (±64) higher in the east and 312 m (±89) higher in the west. Since upslope migration was very rare (east: 8, west: 4), we did not subdivide this pattern.

### 2.4. Analysis

We carried out all the analysis in R 4.0.2 [41]. For our first objective, we visualized the seasonal elevation distribution of species and categorized observable patterns by the positions of breeding and wintering distributions relative to one another. We repeated this process for our second objective, after separating species data by region. For our third objective, we visualized how these migration patterns vary in terms of the elevation species breed at, their diet and habitat guilds, degree of territorial behaviour and body mass. We used χ^2^-tests to examine if the patterns of elevational migration were dependent on attributes like territorial behaviour, diet and habitat of the species. We conducted ANOVAs and post hoc tests (Tukey honestly significant differences) for differences between each migration pattern with respect to breeding elevation and body mass.

To ensure our results were not dependent on the thresholds we chose, we increased the 200 m threshold to 300 m and decreased the 10% overlap threshold (for separating shift and displace migrants) to 5% and repeated all the above analysis. Although some species saw a change in their elevational migration pattern, the overall results relating to intraspecific variation in these patterns and their relation to functional traits remained qualitatively unchanged. We have therefore reported results from the original thresholds and included the results from the sensitivity analysis in the electronic supplementary material.

## 3. Results

**Figure 3.**
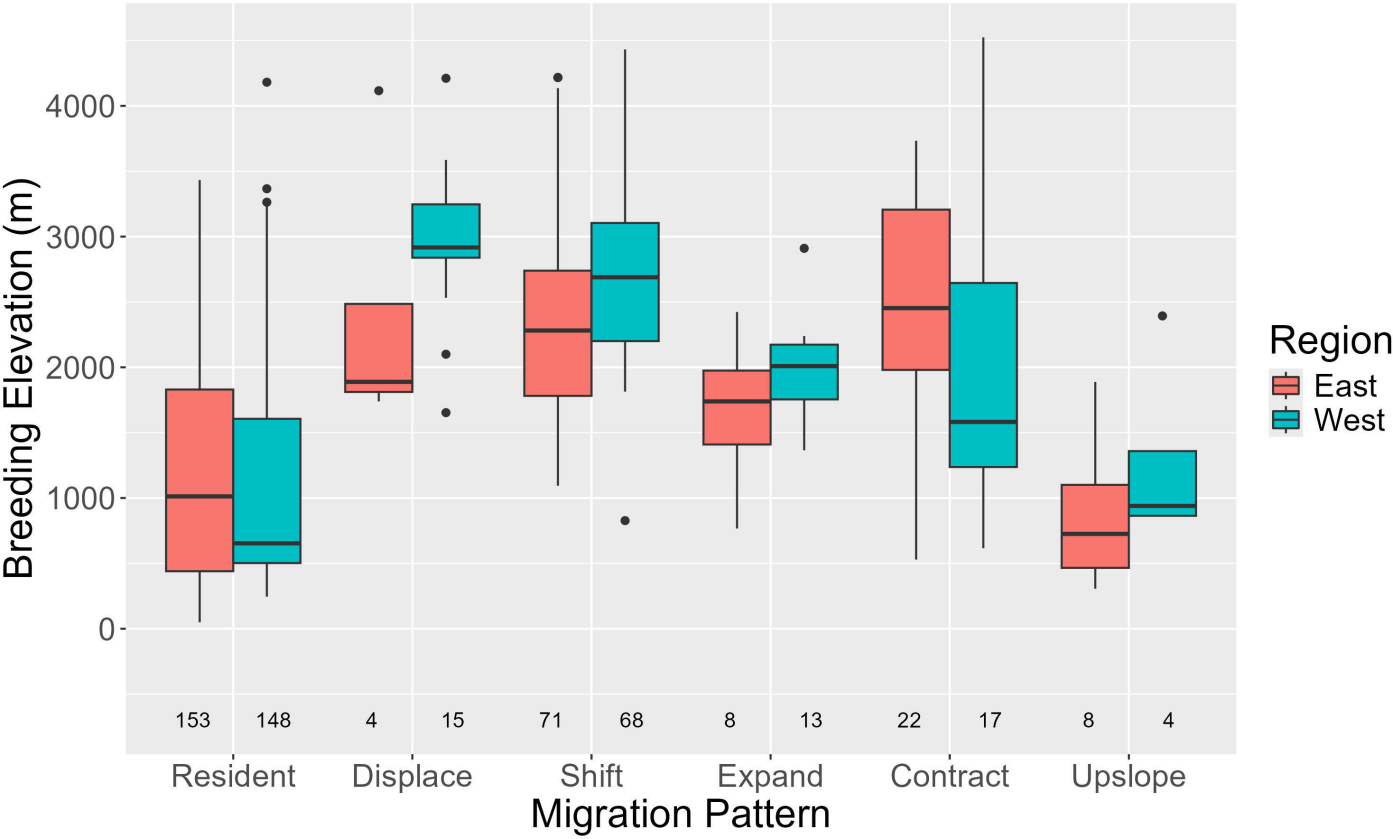
Box plots of the elevational distribution of the breeding ranges (estimated centre of summer elevational range) for Himalayan species by migration pattern with associated sample sizes, separated by region.

We analysed a total of 74 941 checklists (55 483 in the west and 19 458 in the east), extracting winter and summer elevation ranges for 266 eastern and 265 western Himalayan species belonging to 57 families. We found that 58% of eastern and 56% of western species do not show significant elevational movement and were thus considered resident (electronic supplementary material, table S3). However, among species that have some part of their breeding range above 2500 m, 65% of eastern and 74% of western species migrated downslope in the winter. Among the downslope migration patterns, most species (68% of eastern, 60% of western species) showed shift migration. Among shift migrants, all individuals breeding at higher elevations (above the zone of overlap) migrate downslope in the winter. However, individuals that breed in the zone of overlap could potentially be resident. The breeding elevations of species within each migration pattern were largely similar across regions except in the case of displace migrants that bred at higher elevations in the west (figure 3). However, this may be because we found only four species showing displace migration in the east. Upslope migrants and residents bred at similar elevations, which were lower than most types of downslope migrants (figure 3; electronic supplementary material, table S5). Contract migrants bred over a large range of elevations, but on average their breeding elevations were similar to those of expand migrants (electronic supplementary material, table S5). On average, both contract and expand migrants breed higher than residents but lower than shift and displace migrants (figure 3; electronic supplementary material, table S5; *F*_west_ = 50.07, *p*_west_ < 0.01, *F*_east_ = 29.86, *p*_east_ < 0.01).

### 3.1. Intraspecific variation in elevational migration pattern

One hundred fifty-four species analysed were common to both the eastern and western Himalayas, enabling an analysis of intraspecific variation in elevational migration patterns. The mean breeding elevation of these species was similar across the Himalayas, but of these 154 species, 54 (35%) showed different migration patterns across regions. Eighty per cent of eastern and western residents did not change their migration pattern across the Himalayas. Species showing downslope migration patterns were largely downslope migrants irrespective of region (east: 84%; west: 79%), but they varied in their specific downslope pattern. Eight out of ten displace migrants in the west, like Whistler’s Warbler (*Phylloscopus whistlerii*) and Golden Bush Robin (*Tarsiger chrysaeus*), were shift migrants in the east. No contract migrants (of 24) and only three (of 14) expand migrants—Blue Whistling-Thrush (*Myophonus caeruleus*), Common Kingfisher (*Alcedo atthis*) and Great Barbet (*Psilopogon virens*)—showed the same pattern across the Himalayas (electronic supplementary material, table S3).

### 3.2. Functional traits and their relation to elevational migration

#### 3.2.1. Diet

**Figure 4.**
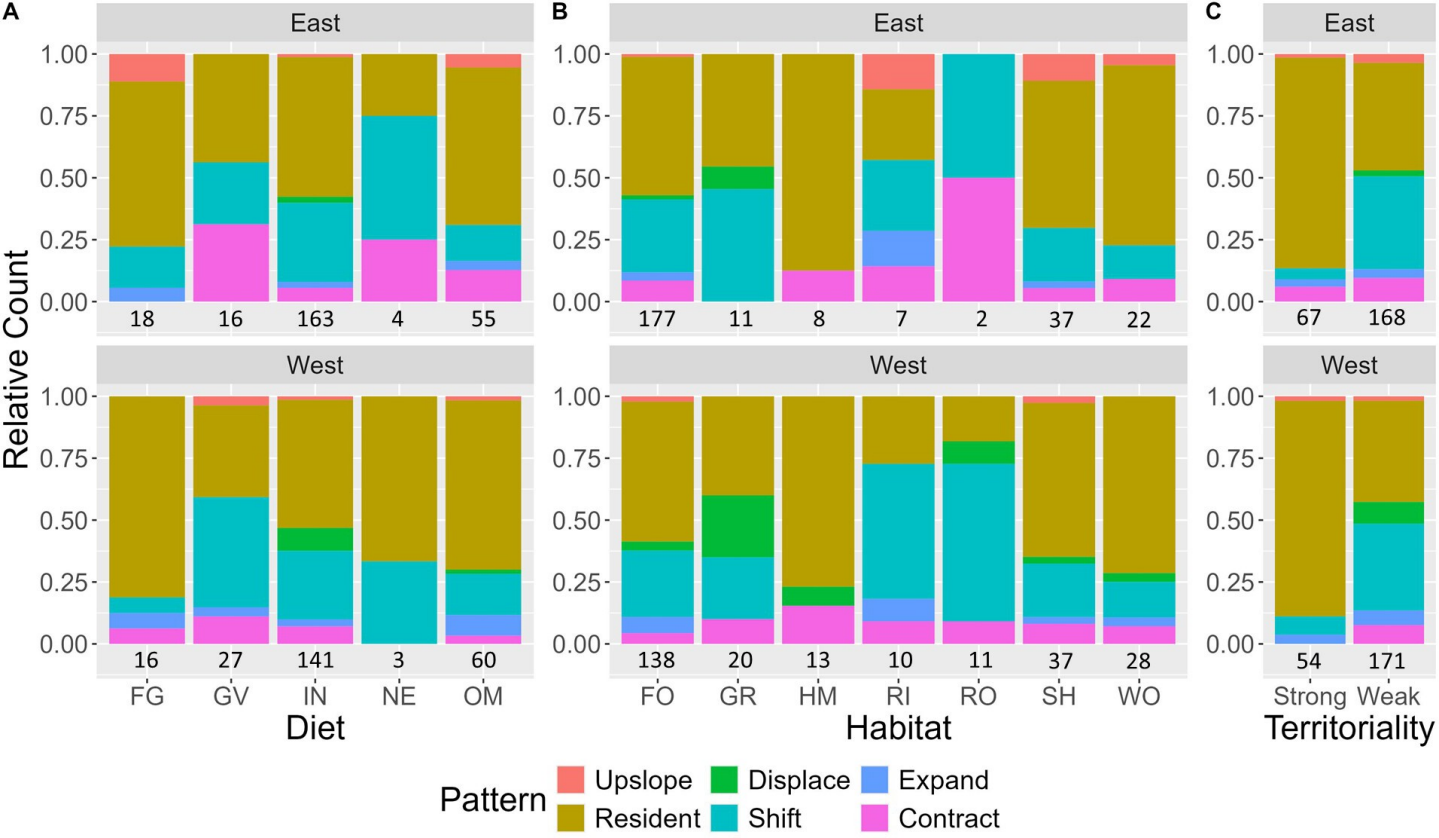
Relative proportions of each migration pattern used by different (A) dietary guilds (FG = frugivore, GV = granivore, IN = invertivore, NE = nectarivore, OM = omnivore), (B) habitat guilds (FO = forest, GR = grassland, HM = human modified, RI = riverine, RO = rock, SH = shrub, WO = woodland) and (C) strength of territoriality in the eastern and western Himalayas. The number of species found in each dietary, habitat and territoriality guild is given below each stacked bar per region.

χ^2^-Tests showed that diet explained certain elevational migration patterns (east: χ^2^ = 34.87, d.f. = 20, *p* < 0.05; west: χ^2^ = 27.69, d.f .= 20, *p* = 0.12). Invertivores were the largest dietary guild in both regions (approx. 60% of species) and were more likely to migrate downslope (electronic supplementary material, table S7). A larger proportion (than expected by chance) of invertivores showed shift migration (figure 4). Seventeen of the 18 species showing displace migration were invertivores (Eurasian Skylark *—Alauda arvensis*—is classified as an omnivore). A larger proportion (than expected by chance) of frugivores and omnivores were residents (figure 4A; electronic supplementary material, table S7). However, there were very few frugivores that breed at higher elevations (>2500 m) and the ones that do, like Black Bulbul (*Hypsipetes leucocephalus*) and Great Barbet (*Psilopogon virens*), largely migrate downslope showing shift and expand migration, respectively. Omnivores were more likely to be resident (in both regions) like Mountain Bulbul (*Ixos mcclellandii*) or contract migrants (in the east) like Eurasian Jay (Garrulus glandarius) and expand migrants (in the west) like Gray Treepie (*Dendrocitta formosae;* electronic supplementary material, table S7). Granivores were more likely to be contract migrants (in both regions) like Chukar (*Leucosticte brandti*) or shift migrants in the west like Pink-browed Rosefinch (*Carpodacus rodochroa;* electronic supplementary material, table S7). Migration patterns associated with nectarivores were difficult to interpret as there were only five truly nectarivorous species (sunbirds) in our dataset (figure 4A).

#### 3.2.2. Habitat

χ^2^-Tests showed that species habitat preferences also played a role in determining certain elevational migration patterns (east: χ^2^ = 39.74, d.f. = 30, *p* = 0.11; west: χ^2^ = 48.24, d.f. = 30, *p* < 0.05). Compared with other habitat guilds, a larger proportion of species inhabiting grasslands, riverine habitats and rocky areas showed downslope migration (figure 4B; electronic supplementary material, table S9). In the west, more grassland birds like Himalayan Rubythroat (*Luscinia pectoralis*) were displace migrants than expected by chance, while birds of rocky habitats like Alpine Chough (*Pyrrhocorax graculus*) were more likely to be shift migrants (electronic supplementary material, table S9). Forest birds had a similar proportion of resident species and downslope migrants; these proportions were similar to what one would expect by chance (electronic supplementary material, table S9). A larger proportion of species (than expected by chance) associated with human-modified habitats, shrublands and woodlands were resident, like Eurasian Magpie (*Pica pica*), Red-billed Leiothrix (*Leiothrix lutea*) and Greater Flameback (*Chrysocolaptes guttacristatus;* figure 4B; electronic supplementary material, table S9).

#### 3.2.3. Territoriality

Territoriality played a role in determining certain elevational migration patterns in both regions (east: χ^2^ = 36.65, d.f. = 5, *p* < 0.01; west: χ^2^ = 37.03, d.f. = 5, *p* < 0.01). A larger proportion of strongly territorial birds were resident, while weakly territorial birds were more likely to be displace, shift and contract migrants (figure 4C; electronic supplementary material, table S11). All displace migrants, irrespective of region, were weakly territorial, while all contract migrants in the west were weakly territorial (figure 4C). When using the difference between a species’ distributional centre in each season to represent the extent of migration, we found that weakly territorial birds (east: 484.18 m; west: 633.96 m) have a significantly greater extent of migration than strongly territorial birds (east: 68.56 m; *t* = 8.04, d.f. = 231.96, *p* < 0.01; west: 72.91 m; *t* = 8.41, d.f = 222.65, *p* < 0.01).

#### 3.2.4. Body mass

**Figure 5.**
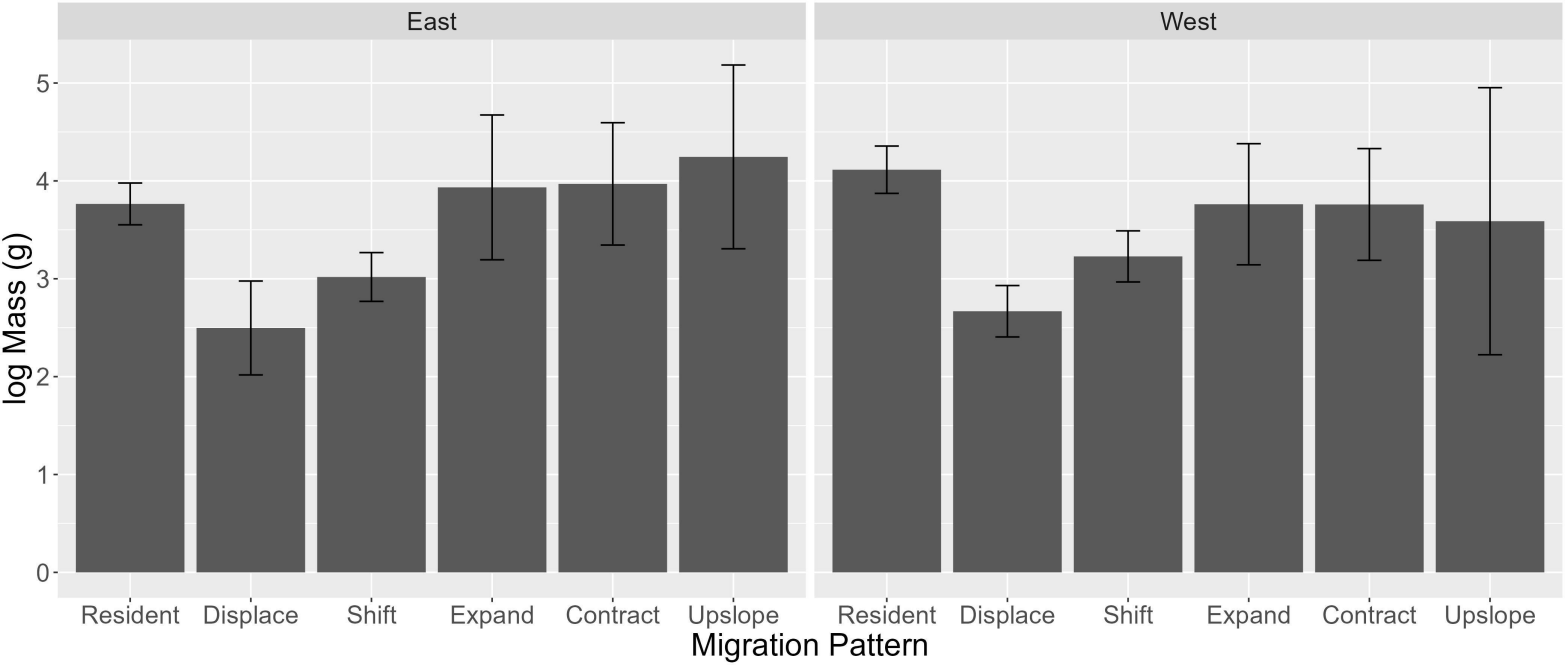
Bar plots of mean log mass for each migration pattern by region. Error bars represent 95% confidence intervals.

Downslope migrants, in general, had a lower log mass than residents (electronic supplementary material, figure S2; *t*_east_ = −3.07, *p*_east_<0.01, *t*_west_ = −5.08, *p*_west_< 0.01). Within downslope migrants, log mass of shift and displace migrants was significantly lower than that of resident species, while expand and contract migrants had similar mean log mass, which was marginally heavier than shift and displace migrants (figure 5; electronic supplementary material, table S13). Displace migrants had a marginally lower mean log mass than shift migrants (figure 5; electronic supplementary material, table S13; *F*_east_ = 4.92, *p*_east_ < 0.01, *F*_west_ = 6.28, *p*_west_ < 0.01).

## 4. Discussion

### 4.1. Diversity of elevational migration patterns exhibited by Himalayan birds

Using a large citizen science dataset, we describe five different patterns in which post-breeding populations of species migrate elevationally in the Himalayas. Because species elevational ranges are bounded within the altitudinal limits of a mountain range, the patterns we describe here are likely to be observed in mountain ranges across the world. While a large number of birds in our dataset were resident, they mostly occurred at low elevations. Between 65 and 74% of species breeding above 2500 m showed downslope elevational migration, similar to rates reported from the region previously ([1,14–16]). Most species that migrate downslope showed substantial overlap at the lower limit of their summer elevation and the upper limit of their winter elevation (i.e. are ‘shift’ migrants). It is unclear to what degree all individuals are truly shifting their ranges downwards seasonally, or whether high-elevation breeders are ‘leap-frogging’ over birds at the lower end of the breeding range, which, at a population or species level, would appear as shift migration. Further studies using mark-recapture techniques or tracking devices could reveal how individual birds migrate, helping tease apart the two possible mechanisms for shift migration [42].

Consequently, species that showed no or minimal overlap between their summer and winter ranges (displace migrants) are the only ones that can be considered obligate migrants with certainty, where every individual of the population migrates downslope. Most displace migrants were flycatchers (*Muscicapidae*), warblers (*Phylloscopidae* and *Scotocercidae*), pipits and wagtails (*Motacillidae*). Williamson & Witt [43] describe an extreme case of displace migration called ‘elevational niche shift migration’, characterized by >2000 m between the lower limit of the breeding elevation and the upper limit of the wintering elevation, with implications for species respiratory physiology. However, we were unable to detect this phenomenon, probably because we did not include elevational migrants that also move significantly latitudinally, like the Bar-headed Goose and Demoiselle Crane. The proportion of displace migrants in the western Himalayas is more than three times that of the eastern Himalayas. This may be because higher elevations in the seasonal west tend to be harsher in the winter and so species breeding at the highest elevations migrate further downslope to track their thermal regimes [15].

Nearly 30% of all downslope migrants showed expand or contract migration patterns, which suggests that several elevational migrants in the Himalayas are partial migrants with intraspecific variation in migration propensity. Expand migration may be seen when climate and food are constrained at the upper limit of a species’ range in the winter, causing individuals with lower physiological tolerances or lower competitive abilities to migrate downslope, while more robust and dominant individuals remain. Similarly, if nesting sites are constrained at the lower limit of a species’ breeding range, some individuals may move higher up to breed and thus show contract migration. Studies have suggested that such intraspecific variation may be driven by body mass, sex, age and dominance hierarchies [10,28,29,44].

### 4.2. Intraspecific variation in elevational migration patterns

We found substantial plasticity associated with downslope migration patterns among populations. While most residents were so over their entire distribution across the Himalayas, many downslope migrants showed different regional patterns. Local site-specific factors, like temperature, habitat availability and competition, may influence species to adapt their migratory behaviour to the available conditions. For example, many species, like the Rufous-gorgeted Flycatcher (*Ficedula stophiata*) and Whistler’s Warbler (*Phylloscopus whistlerii*), are shift migrants in the east but displace migrants in the west. A potential explanation is that the harsher winter climate in the west forces most individuals to migrate further downslope. In the aseasonal east, individuals are known to migrate relatively shorter distances downslope [15], so individuals breeding at the highest elevations do not winter below individuals breeding at the lower elevation limit. Alternatively, individuals breeding at their lower elevation limit overwinter in the same place, while only the birds at the much colder upper limit have to migrate downslope. This suggests that individual migration decisions are likely condition- and context-dependent and reflect a balance between competing for quality breeding territories and maximizing survival prospects during periods of resource limitation [11,45]. These results for individual species need to be taken with caution, given the pitfalls of using thresholds to categorize patterns where species narrowly on either side of the threshold may be categorized differently. However, the larger community level patterns described above were consistent across varying thresholds (electronic supplementary material, table S4). Compared with North America (approx. 25%) and Taiwan (approx. 23%), we found far fewer species that migrate upslope in the winter (approx. 5% in our dataset). Upslope migrants were low-elevation breeders with most breeding <1000 m and migrating <400 m upslope. Whether such a small upward shift can even be considered migration is debatable.

### 4.3. Functional traits and their relation to elevational migration

Invertivores were more likely to show shift and displace migration, presumably tracking climate-sensitive resources, as predicted by the food availability hypothesis. Granivores also track climate-sensitive resources downslope, with a substantial proportion showing contract and shift migration. This may be because some granivores, like finches, irrupt downslope during harsh winters; high-elevation individuals escape excessive snowfall by migrating to lower snow-free elevations, wintering alongside populations at lower elevations. A similar partial migratory strategy is seen in White-winged Snowfinches (*Montifringilla nivalis*) of the Alps, where the southern resident population merges with the northern migratory population in the winter [8]. A larger proportion of frugivores did not show any elevational movement, in contrast to Neotropical studies, where a substantial proportion of elevational migrants were frugivores [3]. This trend was driven by a relative lack of frugivores breeding at high elevations in the Himalayas and hence they were not as affected by harsh winter conditions. As expected, we found omnivorous species more likely to be resident. Because omnivores are typically generalists, they may be able to switch to alternative food resources in winter, thereby obviating the need to migrate to track resources. This may be advantageous as migration can be energetically expensive [46] and lower elevation habitats often have more predators [47]. These results support the findings of Menon *et al*. [15], which found insectivores and granivores to shift further downslope than frugivores and omnivores. Many of the invertivores that overwinter at high elevations are species that can access hibernating insects, such as woodpeckers (*Picidae*) and nuthatches (*Sittidae*) or are known to switch diets and feed on nectar and berries in the winter, such as thrushes (*Turdidae* and *Leiothrichidae*) and scimitar babblers (*Pomatorhinus*) [13,38].

As predicted by the climate constraint hypothesis, species breeding at high elevations in more open habitats like grasslands and rocky environments migrate downslope, with many grassland birds showing strong displace migration patterns. In addition to the less sheltered and harsher microclimate in open habitats potentially affecting a species’ physiological ability to withstand large variations in temperature, species in open habitats largely forage on the ground [23]. Open habitats at higher elevations are covered in snow for many months during winter, so species may find it difficult to access food resources and are hence likely forced to migrate downslope [21]. Species of human-modified habitats were found to be largely resident even at higher elevations. Since most of these species are also omnivores, they may be able to survive on whatever resources are available, including human waste [24,48].

Most strongly territorial species were largely resident, showing very little elevational movement. A large proportion of shift migrants and all displace migrants were weakly territorial. These results give support to the arrival time hypothesis, which assumes that prior residency gives individuals better breeding success by holding high-quality territories from late-arriving individuals [27,49]. Territorial species may prioritize territory defence by migrating very little or not at all, so that they can guard these territories year-round or return quickly; on the other hand, weakly territorial species may prioritize resource availability and migrate downslope in the winter based on local conditions. At the intraspecific level, variation in territorial behaviour can result in species showing expand migration with more territorial individuals, like large males, maintaining their summer territories through the harsh winter, while less territorial sub-adult males and adult females migrate further downslope [21].

We found that both shift and displace migrants were, on average, smaller than resident birds. There is some support of the thermal tolerance hypothesis where larger individuals are better able to thermoregulate and avoid hypothermia because with increasing body size, volume increases more quickly than surface area [29,50]. Another mechanism that has generally been used to explain partial migration and may be applicable in the interspecific context as well is that larger species have better fasting endurance, which allows them to avoid migration risks and stay back at their breeding elevation in the winter [21,29].

## 5. Conclusions

Most of our understanding of elevational migration comes from single-species studies ([8,10,37]; but see [51]), which limits our ability to observe general patterns and make broader generalizations. Our study leverages a large citizen science dataset to define elevational ranges for a large number of species in their breeding and non-breeding seasons along the world’s largest elevation gradient. We find that elevation is not equally sampled by citizen scientists, with birdwatchers frequenting more accessible low-elevation sites. We attempted to control for this variation in sampling effort, too, using a published resampling protocol [15,37]. We acknowledge that, despite these efforts, we may be underestimating the upper elevational limits of many high-elevation species. However, our study does not attempt to describe precise elevation distributions of species, but rather to use citizen science data to provide novel insights into the patterns of elevational migration at the scale of multiple species across an entire mountain range in an under-sampled region. Our study provides some degree of support to the various hypotheses of the potential drivers of elevational migration. However, to understand their relative importance in explaining elevational migration, we require further studies that directly measure the resource availability and the temperatures experienced by species along an elevation gradient. Playback and feeder experiments could also reveal the mechanisms by which territoriality and competition may influence elevational movements [52,53]. Research involving techniques that enable us to characterize and compare the diets of migrating and resident species in their breeding and non-breeding seasons could further help confirm the role of diet in driving elevation migration in birds.

## Data Availability

The data and code from the paper are included in a Dryad repository [54]. Supplementary material is available online [55].
